# Supplementary material for: EPI-SF: essential protein identification in protein interaction networks using sequence features
Source: PeerJ. 2024 Mar 13;12:e17010. doi: 10.7717/peerj.17010 (PMC10944162; doi:10.7717/peerj.17010)
Supplement: Table S1 [file peerj-12-17010-s001.docx]

| **SL. No.** | **AAC Indicators** | **Amino Acid Composition (AAC)** |
| --- | --- | --- |
| 1 | AAC_A | Amino acid composition of Alanine |
| 2 | AAC_C | Amino acid composition of Cysteine |
| 3 | AAC_D | Amino acid composition of Aspartic acid |
| 4 | AAC_E | Amino acid composition of Glutamic acid |
| 5 | AAC_F | Amino acid composition of Phenylalanine |
| 6 | AAC_G | Amino acid composition of Glycine |
| 7 | AAC_H | Amino acid composition of Histidine |
| 8 | AAC_I | Amino acid composition of Isoleucine |
| 9 | AAC_K | Amino acid composition of Lysine |
| 10 | AAC_L | Amino acid composition of Leucine |
| 11 | AAC_M | Amino acid composition of Methionine |
| 12 | AAC_N | Amino acid composition of Asparagine |
| 13 | AAC_P | Amino acid composition of Proline |
| 14 | AAC_Q | Amino acid composition of Glutamine |
| 15 | AAC_R | Amino acid composition of Arginine |
| 16 | AAC_S | Amino acid composition of Serine |
| 17 | AAC_T | Amino acid composition of Threonine |
| 18 | AAC_V | Amino acid composition of Valine |
| 19 | AAC_W | Amino acid composition of Tryptophan |
| 20 | AAC_Y | Amino acid composition of Tyrosine |
