# Supplementary material for: EPI-SF: essential protein identification in protein interaction networks using sequence features
Source: PeerJ. 2024 Mar 13;12:e17010. doi: 10.7717/peerj.17010 (PMC10944162; doi:10.7717/peerj.17010)
Supplement: Table S2 [file peerj-12-17010-s002.docx]

| **SL. No.** | **PCP Indicators** | **Physico-chemical properties (PCP)** |
| --- | --- | --- |
| 1 | PCP_PC | Composition of positively charged residues |
| 2 | PCP_NC | Composition of negatively charged residues |
| 3 | PCP_NE | Composition of neutral charged residues |
| 4 | PCP_PO | Composition of polar residues |
| 5 | PCP_NP | Composition of non-polar residues |
| 6 | PCP_AL | Composition of residues having aliphatic side chain |
| 7 | PCP_CY | Composition of residues having cyclic side chain |
| 8 | PCP_AR | Composition of aromatic residues |
| 9 | PCP_AC | Composition of acidic residues |
| 10 | PCP_BS | Composition of basic residues |
| 11 | PCP_NE_ph | Composition of neutral residues based on pH |
| 12 | PCP_HB | Composition of hydrophobic residues |
| 13 | PCP_HL | Composition of hydrophilic residues |
| 14 | PCP_NT | Composition of neutral residues |
| 15 | PCP_HX | Composition of hydroxylic residues |
| 16 | PCP_SC | Composition of residues having sulphur content |
| 17 | PCP_SS_HE | Composition of residue in secondary structure (Helix) |
| 18 | PCP_SS_ST | Composition of residue in secondary structure (Strands) |
| 19 | PCP_SS_CO | Composition of residue in secondary structure (Coil) |
| 20 | PCP_SA_BU | Composition of residue in solvent accessibility (Buried) |
| 21 | PCP_SA_EX | Composition of residue in solvent accessibility (Exposed) |
| 22 | PCP_SA_IN | Composition of residue in solvent accessibility (Intermediate) |
| 23 | PCP_TN | Composition of tiny residues |
| 24 | PCP_SM | Composition of small residues |
| 25 | PCP_LR | Composition of large residues |
| 26 | PCP_Z1 | Composition of residues having Z1 advanced Physico-chemical properties |
| 27 | PCP_Z2 | Composition of residues having Z2 advanced Physico-chemical properties |
| 28 | PCP_Z3 | Composition of residues having Z3 advanced Physico-chemical properties |
| 29 | PCP_Z4 | Composition of residues having Z4 advanced Physico-chemical properties |
| 30 | PCP_Z5 | Composition of residues having Z5 advanced Physico-chemical properties |
