# Supplementary material for: EPI-SF: essential protein identification in protein interaction networks using sequence features
Source: PeerJ. 2024 Mar 13;12:e17010. doi: 10.7717/peerj.17010 (PMC10944162; doi:10.7717/peerj.17010)
Supplement: Table S3 [file peerj-12-17010-s003.docx]

| **SL. No.** | **PAAC Indicators** | **Pseudo Amino Acid Composition (order 1, traditional) (PAAC)** |
| --- | --- | --- |
| 1 | PAAC1_A | Pseudo amino acid composition of Alanine |
| 2 | PAAC1_C | Pseudo amino acid composition of Cysteine |
| 3 | PAAC1_D | Pseudo amino acid composition of Aspartic acid |
| 4 | PAAC1_E | Pseudo amino acid composition of Glutamic acid |
| 5 | PAAC1_F | Pseudo amino acid composition of Phenylalanine |
| 6 | PAAC1_G | Pseudo amino acid composition of Glycine |
| 7 | PAAC1_H | Pseudo amino acid composition of Histidine |
| 8 | PAAC1_I | Pseudo amino acid composition of Isoleucine |
| 9 | PAAC1_K | Pseudo amino acid composition of Lysine |
| 10 | PAAC1_L | Pseudo amino acid composition of Leucine |
| 11 | PAAC1_M | Pseudo amino acid composition of Methionine |
| 12 | PAAC1_N | Pseudo amino acid composition of Asparagine |
| 13 | PAAC1_P | Pseudo amino acid composition of Proline |
| 14 | PAAC1_Q | Pseudo amino acid composition of Glutamine |
| 15 | PAAC1_R | Pseudo amino acid composition of Arginine |
| 16 | PAAC1_S | Pseudo amino acid composition of Serine |
| 17 | PAAC1_T | Pseudo amino acid composition of Threonine |
| 18 | PAAC1_V | Pseudo amino acid composition of Valine |
| 19 | PAAC1_W | Pseudo amino acid composition of Tryptophan |
| 20 | PAAC1_Y | Pseudo amino acid composition of Tyrosine |
| 21 | PAAC1_lam1 | Sequence correlation factor for lambda 1 |
